# Characteristics and risk factors of children with sleep-disordered breathing in Wuxi, China

**DOI:** 10.1186/s12887-020-02207-5

**Published:** 2020-06-26

**Authors:** Yun Guo, Zhenzhen Pan, Fei Gao, Qian Wang, Shanshan Pan, Shiyao Xu, Yu Hui, Ling Li, Jun Qian

**Affiliations:** 1 Department of Pediatric Respiratory, Wuxi Children’s Hospital, Wuxi Clinical Medical College Affiliated to Nanjing Medical University, No.299-1 at Qingyang Road, Liangxi District, 214023 Wuxi, Jiangsu Province People’s Republic of China; 2 Department of Intensive Care Unit, Wuxi People’s Hospital, Wuxi Clinical Medical College Affiliated to Nanjing Medical University, No.299 at Qingyang Road, Liangxi District, 214023 Wuxi, Jiangsu Province People’s Republic of China

**Keywords:** Sleep, Sleep-disordered breathing, Prevalence, Asthma, Risk factor

## Abstract

**Background:**

Sleep-disordered breathing (SDB) is a common syndrome in children, related to their immune responses, cardiovascular function, and neurocognitive function. This study aimed to determine the prevalence of SDB among children in Wuxi, China, and to evaluate the protective and risk factors of SDB in children.

**Methods:**

A cross-sectional study was conducted on children attending different schools across Wuxi, China, aged 3–14 years old. Of a total of 5630 questionnaires distributed to the parents of the children, 3997 (71.0%) were deemed to be valid. The data on the general sociodemographic factors, children’s allergy and sleep characteristics, and the parents’ sleep characteristics were also collected. The Paediatric Sleep Questionnaire (PSQ) score was used to identify children at high risk of SDB. Odds ratios (ORs) and 95% confidence intervals (CIs) were calculated by logistic regression.

**Results:**

The prevalence of SDB in this cohort was 13.4% (*N* = 534). SDB prevalence significantly differed in children with asthma (28.2% vs. 12.8%, *P* < 0.001), eczema (19.0% vs. 10.0%, *P* < 0.001), urticaria (16.4% vs. 12.9%, *P* < 0.01) and rhinitis (21.4% vs. 10.7%, P < 0.001). No significant differences were found in SDB prevalence with respect to pillow material or quilt material. On multivariate logistic regression analysis, asthma (OR 1.986 (95% CI 1.312–3.007), *P* < 0.01), eczema (OR 1.675 (95% CI 1.377–2.037), *P* < 0.001), rhinitis (OR 1.998 (95% CI 1.635–2.441), suffered from familial sleep sickness (OR 2.416 (95% CI 1.975–2.955), *P* < 0.001) and whose mothers slept for a shorter duration (6 h–8 h: OR 1.370 (95% CI 1.089–1.724), *P* < 0.01; <6 h: OR 3.385(95% CI 2.098–5.461), *P* < 0.001) increased the odds of having SDB. The incidence of SDB significantly decreased with children’s age (6–11 years old: 0R 0.768 (95% CI 0.597–0.989), *P* < 0.05; 12–14 years old: OR 0.691 (95% CI 0.530–0.901), *P* < 0.01).

**Conclusion:**

The results of this study demonstrated that atopic diseases (asthma, eczema, and rhinitis) and family sleep habits were risk factors for SDB in children in Wuxi, China.

## Background

Sleep quality is critical for the growth and development of children [1]. Sleep-disordered breathing (SDB) is a syndrome common in childhood, the spectrum of which ranges from primary snoring to partial or complete airway obstruction, termed obstructive sleep apnea syndrome (OSAS) [2]. It is important to determine the prevalence of SDB in children because of its association with the functioning of various organs, including immune responses, cardiovascular function, and neurocognitive function [2].

The prevalence of SDB has been reported to be 5.1–13.3% in children [3–12] and 8.6–57.8% in asthmatic children [1, 6–8, 11–13]. There are, however, few studies that have explored the prevalence of SDB in China. A study in 2015 reported the proportion of children with sleep quality problems was 8.87% in Beijing [14]. In 2017, the prevalence of SDB increased to 11.6% in the same city [1]. Another multicentric cross-sectional study involving 22,478 children from eight cities in China revealed a prevalence rate of SDB at 12.0% [10]. The prevalence of SDB is found to be related to the ethnic origin of the population [15] and environment [16]. Its prevalence needs to be ascertained and undated in the different regions of the same country and other countries worldwide.

As a fast-developing country, the incidence of atopic diseases, children’s differing sleep environments and parents’ various sleep patterns are evolving in China. However, this may cause SDB, or rather even worsen its occurrence. Thus, the identification of risk factors for SDB is necessary to develop treatments targeted towards these factors.

Recently, a number of different tools have been developed for the diagnosis of SDB. Polysomnography is considered the gold standard method for OSA diagnosis. This technique involves the measurement of various neurophysiological and cardiorespiratory parameters. However, its high cost and limited availability in health centers limit its application in research, especially epidemiological research, which requires large sample sizes. Given these limitations, several sleep questionnaires have been developed. The Paediatric Sleep Questionnaire (PSQ), developed by Chervin et al. [17], has a low cost and a precise cut-off score (> 0.33), and identifies children with OSAS with a sensitivity of 83% and a specificity of 87%. Li et al. confirmed that the PSQ was a successful methodology for the investigation of childhood SDB in Beijing, China, thereby supporting the applicability and generalizability of the PSQ in a large epidemiological survey of childhood SDB in China [18].

The objective of this study was to determine the prevalence of SDB among children in Wuxi, China by using PSQ. Several risk factors were analyzed: atopy diseases (asthma, eczema, urticarial and rhinitis), sleep environment (Pillow material and Quilt material), sleep habits (sleeping position and child independence), parents’ sleep patterns, and familial sleep sickness. Based on these identified risk factors for SDB, the development of treatments is of great importance. This study is therefore necessary for the development of public health utility.

## Methods

### Setting, sampling, and participants

The present study utilized a cross-sectional, randomized, stratified, multistage cluster sampling methodology. According to the statistical formula, $\mathrm{n}=\frac{Z_{\alpha /2}^2P\left(1-P\right)}{\delta^2}$, assuming SDB prevalence was about 10% in children [1, 10, 14], significance at α = 0.05 with Z_α/2_ of 1.96 and acceptable error at 0.1 p, the sample size was calculated as 3457. Allowing for a 20% non-response rate, the final intended sample size was set as 4350.

This study was conducted in Wuxi, Jiangsu, China, divided into 5 districts. Based on the school distribution in each region (20.6, 27.7, 17.9, 17.9 and 15.9%), the regional sample size was determined as 896, 1205, 779, 779 and 692, respectively. The respondents were recruited from 9 kindergartens, 10 primary schools, and 9 middle schools, and encompassed children aged 3–14 years. Base on the sample size of each group and school scale, the selection of the school and each grade among the same educational institutions was based on computer-generated random numbers [18].

### Questionnaire

General sociodemographic data were collected, including gender, age, weight (kg), and height (cm). Weight and height were measured by school doctors and investigating doctors upon return of questionnaires. Body mass index (BMI) was calculated as weight [kg]/height [m^2^]. The survey included children’s allergy information such as diagnosis of asthma, diagnosis of eczema, diagnosis of urticaria and diagnosis of rhinitis. In addition, data were collected on sleep characteristics, namely pillow material, quilt material, sleeping position, and sleep environment. Data on family sleep characteristics were also collected, namely parents’ sleeping rules, bedtime, and daily sleep duration, as well as familial sleep sickness.

Atopic disease (asthma, eczema, urticaria, and rhinitis) diagnoses were re-confirmed by a doctor. The diagnosis of asthma was done in accordance with the guidelines for the diagnosis and optimal management of asthma in children [19]. Guidelines for diagnosis and treatment of allergic rhinitis and Clinical Practice Guidelines for diagnosis and treatment of allergic rhinitis in pediatrics were used to diagnose rhinitis [20, 21]. Those with urticaria and eczema were diagnosed previously, and our doctor confirmed the diagnoses by reviewing previous electronic or paper medical records.

### Sleep and respiratory data

In this study, the PSQ was completed by the parents of each child to assess sleep-associated respiratory symptoms [17]. The PSQ is a multi-page questionnaire that consists of closed question-items and several queries on pediatric sleep disorder symptoms. This questionnaire has frequently been used for research [1, 11, 12, 14, 16, 18, 22–25]. It comprises 22 questions on snoring, sleepiness, and behavioral problems. The three possible responses to each question were “yes,” “no,” and “don’t know”; the questionnaire was deemed invalid if any question was answered with “don’t know.” The total number of “yes” answers was calculated, and was divided by the total number of answers. Children with a score of > 0.33 were considered to be at high risk of SDB [17]. To remove the bad inference of flu or other acute infection on sleep, the questionnaire needed parents to assess children’s sleep actions during the past month and sleep conditions during infection was excluded since these may not have been typical.

### Data inclusion and exclusion

The questionnaire was sent to parents by the school teacher and the parents took it home to complete it. The questionnaire was collected 1 week later. After recovery, the missing values of more than 10% of the items in the PSQ were excluded, and the remaining valid questionnaires were signed by a supervisor.

### Data processing

EpiData V.3.1 software (The EpiData Association, Odense, Denmark) was used for data entry. Two staff members independently entered the questionnaire data, and the quality of these data was checked by a third member of the staff. Coding and double entry of the questionnaire data were independently carried out by two professional data-entry staff.

### Statistical analysis

SPSS Statistics 23.0 for Win10 (IBM, New York, U.S.) was used for data processing. Normally distributed data are expressed as mean ± standard deviation. Differences between groups were analyzed using the t-test if the group variances were homogeneous or the Mann-Whiney U test if the group variances were heterogeneous. Non-normally distributed data are represented as the median and interquartile range (IQR). Pearson’s chi-squared test or Fisher’s exact test was used to analyze these data. The Chi-square test, t-test, and Mann-Whiney U test were performed to select possible risk factors for SDB. Stepwise logistic regression was performed to reduce the questionnaire items to the set of items that were risk or protect factors in SDB. A *P* value of 0.05 was selected a priori as the criterion for item retention and finally filtered to six variables. These six variables were analyzed by multivariate logistic regression to explore the risk factors for SDB. Odds ratios (ORs) and 95% confidence intervals (CIs) were calculated by logistic regression. A *P*-value < 0.05 was considered statistically significant.

## Results

In total, 5630 questionnaires were distributed to the parents of these children and 4599 questionnaires were returned, with a response rate of 81.7%, fulfilling more than the expected sample size of 4350. Unreturned questionnaires were considered to be due to the following factors: some children live with grandparents, and their grandparents do not have the ability to complete the questionnaire; the questionnaire was taken home by the parent to complete, and some parents may have forgotten to complete or return it. There were 602 questionnaires that were excluded because of incompleteness and false information. Finally, a total of 3997 (71.0%) were deemed to be valid. Sample characteristics difference between complete responders and incomplete responders were not significant, they were described in supplementary Table 1.

In total, 3997 children were included in this study, 47.7% (1906) of whom were male. The mean age and BMI of the children were 9.50 ± 3.12 years and 16.64 (IQR 14.99–18.84) kg/m^2^, respectively. Table 1 shows the demographic characteristics of the study population, categorized into SDB and non-SDB groups according to PSQ score. The overall prevalence of SDB was 13.4% (*N* = 534). SDB prevalence significantly differed between allergy groups: 28.2% of children with asthma had SDB (37/131, χ^2^ = 25.922, *P* < 0.001), 19.0% of children with eczema had SDB (283/1487, χ^2^ = 65.802, P < 0.001), 16.4% of children with urticaria had SDB (78/475, χ^2^ = 4.364, *P* < 0.05), and 21.4% of children with rhinitis had SDB (215/1003, χ^2^ = 75.442, P < 0.001). No significant differences were found between children with SDB and those without SDB with respect to pillow material or quilt material. The prevalence of SDB was significantly higher in children who slept in the prone position (18.3%), than in those who slept in the lateral position (12.8%) or supine position (13.0%) (χ^2^ = 8.007, *P* < 0.05). Children who share a bed with their parents while sleeping tended to show a higher prevalence of SDB compared to those who slept alone (15.1% versus 12.7 and 12.4%), although the differences between these groups were not significant (χ^2^ = 5.651, *P* > 0.05).

**Table 1:** Sample characteristics

Characteristic	Total (*n* = 3997)	SDB (n = 534, 13.4%)	NO SDB (*n* = 3463, 86.6%)	Group differences	P
Age in years (mean SD)	9.50 ± 3.12	9.00 ± 3.17	9.58 ± 3.11	t = −3.974	0.000*
Male(%)	1906 (47.7)	271 (14.2)	1645 (85.8)	χ^2^ = 2.318	NS
Height (cm) median (min-max)	139.00 (120.00–152.00)	135.00 (104.00–150.00)	140.00 (120.00–153.00)	Z = -3.498	0.000*
Weight (kg) median (min-max)	31.00 (22.00–42.50)	30.00 (21.00–43.00)	32.00 (22.50–43.00)	Z = -2.879	0.004
BMI median (min-max)	16.64 (14.99–18.84)	16.43 (14.88–19.00)	16.65 (15.00–18.85)	Z = -0.787	NS
Asthma	131	37 (28.2)	94 (71.8)	χ^2^ = 25.922	0.000*
Eczema	1487	283 (19.0)	1204 (81.0)	χ^2^ = 65.802	0.000*
Urticaria	475	78 (16.4)	397 (83.6)	χ^2^ = 4.364	0.044†
Rhinitis	1003	215 (21.4)	788 (78.6)	χ^2^ = 75.442	0.000*
Sleep duration/night, hours median (min-max)	9.00 (8.08–9.50)	9.00 (8.00–9.50)	9.00 (8.10–9.50)	Z-0.829	NS
Day naps, hours median (min-max)	0.00 (0.00–1.00)	0.00 (0.00–1.50)	0.00 (0.00–1.00)	Z = -0.787	0.022
Pillow material	3864	520 (13.5)	3344 (86.5)	χ^2^ = 8.908	NS
Buckwheat	765 (19.8)	95 (12.4)	670 (87.6)		
Silk	153 (4.0)	28 (18.3)	125 (81.7)		
Down/ Feather	337 (8.7)	40 (11.9)	297 (88.1)		
Sponge	812 (21.0)	118 (14.5)	694 (85.5)		
Chemical fiber	616 (15.9)	69 (11.2)	547 (88.8)		
Others	1181 (30.6)	170 (14.4)	1011 (85.6)		
Quilt material	3877	521 (13.4)	3356 (86.6)	χ^2^ = 9.745	NS
Cotton	2497 (64.4)	324 (13.0)	2173 (87.0)		
Down/ Feather	246 (6.3)	43 (17.5)	203 (82.5)		
Silk	851 (21.9)	121 (14.2)	730 (85.8)		
Wool	78 (2.0)	6 (7.7)	72 (92.3)		
Blanket	29 (0.7)	2 (6.9)	27 (93.1)		
Chemical fiber	112 (2.9)	19 (17.0)	93 (83.0)		
Others	64 (1.7)	6 (9.4)	58 (90.6)		
Sleeping position	3997	534 (13.4)	3463	χ^2^ = 8.007	0.018†
Supine	1275 (31.9)	166 (13.0)	1109 (87.0)		
Lateral	2378 (59.5)	305 (12.8)	2073 (81.2)		
Prone	344 (8.6)	63 (18.3)	281 (81.7)		
Sleep environment	3997	534 (13.4)	3463	χ^2^ = 5.396	NS
Shares a bed	1307 (32.7)	198 (15.1)	1109 (84.9)		
Shares a bedroom	631 (15.8)	80 (12.7)	551 (87.3)		
Sleep alone	2059 (51.5)	256 (12.4)	1803 (87.6)		

Data were presented as mean ± SD, median, min-max or n(%) unless otherwise stated. P < 0.05 was considered statistically significant difference. NS, no significant difference; SDB, sleep disorder breathing; SD, standard deviation; min-max, minimum and maximum interquartile range

A child’s risk of SDB was found to be associated with the sleep conditions, characteristics, patterns and habits of their family (Table 2). A significantly higher proportion of children whose fathers and mothers both exhibited irregular sleeping patterns suffered from SDB compared to those whose fathers and mothers both exhibited regular sleeping patterns (χ2 = 50.994, *P* < 0.001; χ2 = 52.101, P < 0.001, respectively). The prevalence of SDB was significantly higher in children whose parents went to sleep later and had a shorter duration of sleep (*P* < 0.05). The prevalence of SDB was significantly higher in children from families with a history of familial sleep sicknesses than in those from normal families (χ^2^ = 99.219, *P* < 0.001).

**Table 2:** Family sleep characteristics

Characteristic	Sample (n = 3997)	SDB (n = 534)	NO SDB (n = 3463)	Group differences	*P* values
Father’s sleep patterns	3997	534 (13.4)*	3463	χ^2^ = 50.994	0.000*
Irregular	817	163 (20)	654		
Regular	2486	318 (12.8)	2168		
Very regular	694	53 (7.6)	641		
Mother’s sleep patterns	3997	534 (13.4)*	3463	χ^2^ = 52.101	0.000*
Irregular	385	90 (23.4)	295		
Regular	2833	381 (13.4)	2452		
Very regular	779	63 (8.1)	716		
Father’s bedtime	3997	534 (13.4)*	3463	χ^2^ = 30.033	0.000*
21–22	1247	118 (9.5)	1129		
22–24	2440	355 (14.5)	2085		
After 24	310	61 (19.7)	249		
Mother’s bedtime	3997	534 (13.4)*	3604	χ^2^ = 26.946	0.000*
21–22	2316	265 (11.4)	2051		
22–24	1572	241 (15.3)	1331		
After 24	109	28 (25.7)	81		
Father’s sleep duration	3997	534 (13.4)*	3463	χ^2^ = 14.940	0.001*
>8 h	686	68 (9.9)	618		
6 h–8 h	3093	423 (13.7)	2670		
<6 h	218	43 (19.7)	175		
Mother’s sleep duration	3997	534 (13.4)*	3463	χ^2^ = 27.318	0.000*
>8 h	1091	113 (10.4)	978		
6 h–8 h	2790	390 (14.0)	2400		
<6 h	116	31 (26.7)	85		
Familial sleep sickness	637 (20.9)	199 (23.8)*	638	χ^2^ = 99.219	0.000*

Data were presented as n(%) unless otherwise stated. *P* < 0.05 was considered statistically significant difference. *SDB* sleep disorder breathing

The Chi-square test, t-test and Mann-Whiney U test were performed to select possible risk factors for SDB. According to pervious results, 13 items were possible risk factors for SDB. On univariate logistic regression analysis, SDB incidence decreased with age (6–11 years old: 0R 0.788 (95% CI 0.619–1.002), *P* > 0.05; 12–14 years old: OR 0.658(95% CI 0.513–0.844), *P* < 0.01). Atopic diseases including asthma (OR 2.668 (95% CI 1.803–3.948), *P* < 0.001), eczema (OR 2.115 (95% CI 1.760–2.542), P < 0.001), urticaria (OR 1.321 (95% CI 1.017–1.717), P < 0.001) and rhinitis (OR 2.288 (95% CI 1.891–2.768) increased the odds of having SDB. Sleep patterns (irregular) and habits (sleep less than 8 h or sleeps late) of their parents were predictors of SDB. Familial sleep sickness was also a risk factor for SDB (OR 2.630 (95% CI 2.164–3.198), *P* < 0.001) (Supplementary Table 2).

The variables including age, asthma status, eczema status, rhinitis status, mother’s sleep duration status and familial sleep sickness status were finally included in the multiple logistic regression analysis (*P* < 0.05). SDB incidence significantly decreased with age (6–11 years old: 0R 0.768 (95% CI 0.597–0.989), P < 0.05; 12–14 years old: OR 0.691 (95% CI 0.530–0.901), *P* < 0.01). A number of atopic diseases were predictors of SDB, namely asthma (OR 1.986 (95% CI 1.312–3.007), *P* < 0.01), eczema (OR 1.675 (95% CI 1.377–2.037), *P* < 0.001) and rhinitis (OR 1.998 (95% CI 1.635–2.441). Children who suffered from familial sleep sicknesses (OR 2.416 (95% CI 1.975–2.955), P < 0.001) and whose mothers slept for a shorter duration (6 h–8 h: OR 1.370 (95% CI 1.089–1.724), P < 0.01; <6 h: OR 3.385(95% CI 2.098–5.461), P < 0.001) also increased the odds of having SDB (Table 3).

**Table 3:** Risk Factors for Sleep-disordered Breathing in children

Characteristic	Univariate analysis		Multivariate analysis	
	OR(95% CI)	*P* values	OR(95% CI)	*P* values
Age in years		0.004*		0.022†
3–5	1		1	
6–11	0.788 (0.619–1.002)	0.052	0.768 (0.597–0.989)	0.041†
12–14	0.658 (0.513–0.844)	0.001*	0.691 (0.530–0.901)	0.006*
Asthma		0.000*		0.001*
No	1		1	
Yes	2.668 (1.803–3.948)		1.918 (1.267–2.905)	
Eczema		0.000*		0.002*
No	1		1	
Yes	2.115 (1.760–2.542)		1.735 (1.422–2.117)	
Rhinitis		0.000*		0.000*
No	1		1	
Yes	2.288 (1.891–2.768)		2.039 (1.662–2.503)	
Mother’s sleep duration		0.000*		0.000*
>8 h	1		1	
6 h–8 h	1.406 (1.126–1.756)	0.003*	1.369 (1.083–1.729)	0.009*
<6 h	3.156 (2.003–4.975)	0.000*	3.424 (2.102–5.577)	0.000*
Familial sleep sickness		0.000*		0.000*
No	1		1	
Yes	2.630 (2.164–3.198)		2.339 (1.903–2.874)	

The data from multivariate analysis were after adjusting for age, asthma status, eczema status, rhinitis status, mother’s sleep duration status and familial sleep sickness status. *P* < 0.05 was considered statistically significant difference. CI, confidence interval; OR, Odds ratios

## Discussion

China is a rapidly industrializing developing country with increasing attention on childhood sleep health. Although there were data of SDB being an epidemic in Beijing, China, childhood sleep habits and SDB-related symptoms of different cities may be affected by regions and a survey of other cities is needed. Clear and complete epidemic statistics resulted in a growing number of people learning the importance of SDB, thereby decreasing its debilitating and costly effects in the future.

The main findings of the present study were that SDB prevalence in children aged 3–14 years in Wuxi, China was 13.4%. while children with asthma, eczema, urticarial or rhinitis showed an increased prevalence of SDB as compared to unaffected children. Family sleep habits (shorter maternal sleep duration, familial sleep sickness, and irregular parental sleep patterns) also resulted in a high prevalence of SDB. On multivariate logistic regression analysis, we found asthma, eczema, rhinitis, familial sleep sickness and having mothers who slept for a shorter duration increased the odds of having SDB. However, SDB incidence significantly decreased with age.

This was a cross-sectional study with a total of 3997 children aged 3–14 years. The overall SDB prevalence in this study was 13.4%. In a cohort of primary school students in Japan, the prevalence of SDB varied from 8.5 to 11.3% [26]. Furthermore, in a study of a random sample of 20,152 children from eight Chinese cities, the prevalence of childhood habitual snoring was 12.0% [4]. A cross-sectional telephone questionnaire survey in which 3047 6 to 12-year-old children, with no apparent health problems, reported a habitual snoring prevalence of 10.9% [27]. The prevalence of SDB in this study being higher than that reported in previous studies may be related to the population subsets, broad age range and distinctive environments.

We noticed that SDB incidence significantly decreased with age and therefore, age was a protect factor for SDB. SDB was manifested in 16.6% of children aged 3–5 years, 13.6% of children aged 6–11 years and 11.6% of children aged 12–14 years. The relationship between age and SDB was according to diseases, allergen exposure, study-induced stress, sleep environment and so on. Children aged 3–5 years are susceptible to suffering from respiratory infection, and repeated infection may influence the SDB prevalence. However, the incidence of tonsillar and adenoidal hypertrophy may also lead to the peak prevalence at this age group [28]. Moreover, diagnosing asthma in children aged 3–5 years is difficult, resulting to untreated asthma which will affect children’s sleep [12]. For children aged 6–11 years, the number of respiratory infections is reduced and their SDB always associated with allergic diseases [12, 29, 30]. For children aged 12–14 years, mainly enrolled in middle schools, given heavy academic pressure and graduating pressure, in addition to a heavy study load and homework makes them suffer from sleep loss [31]. However, children aged 12–14 years old always sleep alone and parents cannot observe their sleep conditions very well which may affect SDB prevalence.

Among other demographic correlates, we did not identify an association between SDB symptoms and gender, correlating with the findings of a previous study [2]. An association between SDB symptoms and BMI was also not identified in this study. This is in contrast to the findings of some previous studies in children, in which obesity was identified as an important risk factor for SDB [32–34]. Obesity likely contributes to SDB by increasing fat deposition in the subcutaneous fat of the neck and other soft tissue structures, and reducing lung volume [35]; we did not, however, get a positive result possibly because the proportion of obese people in the group is low (data not shown).

The relationship between atopic diseases and SDB has gained attention in recent years. In this study, SDB prevalence significantly increased in asthmatic children than non-asthmatic children (28.2% vs. 12.8%) and asthma was identified as a strong factor for SDB. We found children with asthma more often suffer from “heavy or loud breathing”, “trouble breathing or struggle to breathe”, “stop breathing during the night”, “wake up feeling unrefreshed in the morning” and “wake up with headaches in the morning” than normal children (data not shown). Several studies provide evidences that SDB prevalence is highly associated with asthma. A systematic review of 17 studies involving 45,155 children found that SDB was significantly more prevalent in children with asthma than non-asthmatics (23.9% vs. 16.7%) [5]. Asthma may impair sleep via several mechanisms. Asthma may affect sleep by changing the circadian rhythm [36, 37]. A common inflammatory pathway of the airway may also link asthma and SDB [5]. Increased inflammation and airway resistance affects the airways and causes decreased airway flow rates during sleep. Increased bronchial hyper-reactivity during sleep may be related to allergen exposure, airway cooling, hormonal changes, and parasympathetic tone [38, 39]. These mechanisms provide evidences that poor asthma control and concomitant presence of rhinitis are associated with high risk for SDB in children [12]. In this study, about 18.3% (24/131) of asthmatics were not well controlled, and about 57.3% (75/131) of asthmatics had a concomitant presence of rhinitis.

We also found a high frequency of SDB with rhinitis (21.4% vs. 10.7%) and rhinitis was one of the risk factors for OSA. Sleep disorder symptoms in rhinitis were different from those present in the asthmatics. Children with rhinitis more often suffer from “snoring”, “heavy or loud breathing”, “breathing through the mouth” and “have a dry mouth on waking up in the morning” than do normal children (data not shown), these different symptoms that indicate the mechanism through which rhinitis affect sleep were distinct. Nasal congestion caused by inflammation of the nasal mucosa may induce oral breathing, sleep disruption, and increased fatigue [40]. High presence pf adenoid hypertrophy in children with rhinitis may also result in sleep problems [41, 42]. Hence, nasal congestion and adenoidal hypertrophy are treatable causes of SDB in children [9]. A 6-week treatment with intranasal budesonide effectively reduced the severity of mild OSAS and the magnitude of the underlying adenoidal hypertrophy, and this effect persisted for at least 8 weeks after cessation of therapy [43]. Our study did not show the relationship between nasal steroids and SDB prevalence due to the missing values of more than 30%. Within the present values, the usage of nasal steroids for rhinitis was low, and this may increase positive findings of SDB.

SDB prevalence significantly increased in eczema (19.0%) and urticaria (16.4%) in our study. Between eczema and urticaria, eczema was a higher risk factor for SDB. Skin allergic-related nocturnal itching and the subsequent scratch response are thought to induce poor sleep initiation and frequent and prolonged awakenings throughout the night [44]. A limitation of this study was the unknown proportion of children who had rashes or urticaria at time of this questionnaire which may affect SDB prevalence.

This study also assessed the relationship between sleep environment and SDB prevalence. However, we did not identify an association between pillow or quilt material and SDB prevalence. As for bed-sharing, we found children who shared a bed with their parents tended to show a higher prevalence of SDB as compared to those who shared a bedroom with their parents but slept on a separate bed or slept alone (15.1, 12.7 and 12.4%, respectively). Bed-sharing has been associated with several sleep problems in children, including frequent waking up throughout and/or an increased length of time spent awake at night, experiencing nightmares, and a shorter night-time sleep duration [45, 46]. In a previous study, sleep problems were more common in children who shared a bed or bedroom with their parents in China [47]. Our data also showed that SDB prevalence in children who shared beds with their parents was higher, however, these differences were not significant.

In this study, the prevalence of SDB was higher in children who slept in the prone position (18.3%) than in those who slept in the lateral position (12.8%) or supine position (13.0%). Children found it easier to sleep in the prone position than adults; the prone and supine positions exhibit numerous differences in the control of the cardiovascular, respiratory, and thermoregulatory systems [48]. The prone sleeping position has been associated with an increased risk of infant death [49]; however, the relationship between this sleeping position and SDB prevalence remains unclear.

We noticed that SDB prevalence was also associated with family sleep conditions. In addition, familial sleep sickness was identified as a risk factor for SDB (OR = 2.202). Family history has long been identified as a strong risk factor for OSAS in individuals of various ethnicities [35, 50, 51], our data supports and extends these observations.

In addition, we evaluated whether a child’s risk of SDB was affected by their parents’ sleep patterns. It was shown that SDB was more prevalent in children whose parents exhibited irregular sleep patterns than in those whose parents exhibited regular sleep patterns. Furthermore, SDB was more prevalent in children whose parents went to sleep later and had a shorter sleep duration. In cases where a child’s parents go to bed at a later time, their bedtime may also be delayed, resulting in shorter nocturnal sleep duration, greater nocturnal variability, and ultimately nocturnal sleep problems [52, 53]. Although mother’s sleep duration was identified as a risk factor for SDB, it is considered that parents who sleep late may notice their children’s sleep conditions easily, which could have a false higher proportion of SDB. The relationship between SDB and parents’ sleep patterns needs further verification.

This study has some limitations that should be noted. First, given the cross-sectional nature of this study, we were unable to establish a cause and effect relationship. In this study, SDB diagnosis was based on a parentally-completed questionnaire. Thus, whether a child snored or stopped breathing during the night was subjective, therefore, the prevalence of SDB in this cohort may have been over or under-estimated, which may have affected the reported SDB prevalence. For disease-related studies, the usage of nasal steroids for rhinitis and the proportion of children who had rashes or urticaria at time of this questionnaire were unknown, which may affect SDB prevalence.

## Conclusion

In summary, the prevalence of SDB in children in Wuxi, China was 13.4%. Atopic diseases (asthma, eczema, and rhinitis), shorter maternal sleep duration, and familial sleep sickness were found to be risk factors for SDB, whereas increased age and regular parental sleep patterns were protective factors. Clinicians should investigate the atopic diseases related to SDB and advise parents to sleep earlier and engage in more regular treatments and counselling.

## Supplementary information

**Additional file 1: Supplementary Table 1.** Sample characteristics. **Supplementary Table 2.** Risk Factors for Sleep-disordered Breathing in children.

## Data Availability

The datasets generated and/or analysed during the current study are not publicly available due data do not have consent from all patients to share their information online but are available from the corresponding author on reasonable request.

## Abbreviations

SDB: Sleep-Disordered Breathing
ORs: Odds Ratios
CIs: Confidence Intervals
OSAS: Obstructive Sleep Apnoea Syndrome
PSQ: Paediatric Sleep Questionnaire
BMI: Body Mass Index
IQR: Interquartile range
