# Supplementary material for: Characteristics and risk factors of children with sleep-disordered breathing in Wuxi, China
Source: BMC Pediatr. 2020 Jun 26;20:310. doi: 10.1186/s12887-020-02207-5 (PMC7318769; doi:10.1186/s12887-020-02207-5)
Supplement: Supplementary file 1 — Additional file 1: Supplementary Table 1. Sample characteristics. Supplementary Table 2. Risk Factors for Sleep-disordered Breathing in children. [file 12887_2020_2207_MOESM1_ESM.docx]

Supplementary table 1 Sample characteristics

| Characteristic | Total | Complete responders | Incomplete responders | Group differences | P |
| --- | --- | --- | --- | --- | --- |
| Age in years (mean, SD) | 9.51±3.12 | 9.50±3.12 | 9.54±3.10 | t=-.214 | 0.831 |
| Male(%) | 2172(47.3) | 1906(47.7) | 266(44.6) | χ^2^=1.942 | 0.163 |
| Height(cm)  (median, min-max) | 138.00  (120.00-152.00) | 139.00  (120.00-152.00) | 136.00  (120.00-149.00) | Z=-1.059 | 0.290 |
| Weight(kg)  (median, min-max) | 31.00  (22.50-42.00) | 31.00  (22.00-42.50) | 29.90  (23.68-39.79) | Z=-0.577 | 0.564 |
| BMI  (median, min-max) | 16.67  (15.00-18.90) | 16.64  (14.99-18.84) | 17.30  (14.79-19.99) | Z=-1.848 | 0.065 |
| Asthma | 151 | 131(3.3) | 20(3.6) | χ^2^=0.187 | 0.665 |
| Urticaria | 551 | 475(11.9) | 76(13.8) | χ^2^=1.699 | 0.209 |
| Sleep duration/night, hours (median, min-max) | 9.00  (8.06-9.50) | 9.00  (8.08-9.50) | 9.00  (8.50-9.50) | Z=-1.192 | 0.233 |
| Day naps, hours  (median, min-max) | 0.0  0(0.00-1.17) | 0.00  (0.00-1.00) | 0.00  (0.00-2.00) | Z=-1.799 | 0.072 |
| Pillow material | 4418 | 3864 | 554 | χ^2^=4.146 | 0.529 |
| Buckwheat | 884(20.0) | 765(19.8) | 119(21.5) |  |  |
| Silk | 174(3.9) | 153(4.0) | 21(3.8) |  |  |
| Down/ Feather | 375(8.5) | 337(8.7) | 38(6.9) |  |  |
| Sponge | 925(20.9) | 812(21.0) | 113(20.4) |  |  |
| Chemical fiber | 697(15.8) | 616(15.9) | 81(14.6) |  |  |
| Others | 1363(30.9) | 1181(30.6) | 182(32.9) |  |  |
| Quilt material | 4439 | 3877 | 562 | χ^2^=8.342 | 0.214 |
| Cotton | 2878(64.8) | 2497(64.4) | 381(67.8) |  |  |
| Down/ Feather | 273(6.2%) | 246(6.3) | 27(4.8) |  |  |
| Silk | 974(21.9) | 851(21.9) | 123(21.9) |  |  |
| Wool | 85(11.9) | 78(2.0) | 7(1.2) |  |  |
| Blanket | 31(0.7) | 29(0.7) | 2(0.4) |  |  |
| Chemical fiber | 130(2.9) | 112(2.9) | 18(3.2) |  |  |
| Others | 68(1.5) | 64(1.7) | 4(0.7) |  |  |
| Sleeping position | 4576 | 3997 | 579 | χ^2^=2.310 | 0.315 |
| Supine | 1464(32.0) | 1275(31.9) | 189(32.6) |  |  |
| Lateral | 2729(59.6) | 2378(59.5) | 351(60.0) |  |  |
| Prone | 383(8.4) | 344(8.6) | 39(6.7) |  |  |
| Sleep environment | 4581 | 3997 | 584 | χ^2^=5.006 | 0.082 |
| Shares a bed | 1519(33.2) | 1307(32.7) | 212(36.3) |  |  |
| Shares a bedroom | 731(16.0) | 631(15.8) | 100(17.1) |  |  |
| Sleep alone | 2331(50.9) | 2059(51.5) | 272(46.6) |  |  |

Data were presented as mean±SD, median, min-max or n(%) unless otherwise stated. P <0.05 was considered statistically significant difference. SDB, sleep disorder breathing; SD, standard deviation; min-max, minimum and maximum interquartile range. Items eczema and rhinitis were excluded due to the missing balues more than 30%.

Supplementary table 2 Risk Factors for Sleep-disordered Breathing in children

| Characteristic | Univariate analysis | |
| --- | --- | --- |
|  | OR(95% CI) | P values |
| Age in years  3-5  6-11  12-14 | 1  0.788(0.619-1.002)  0.658(0.513-0.844) | 0.004*  0.052  0.001* |
| Asthma  No  Yes | 1  2.668(1.803-3.948) | 0.000* |
| Eczema  No  Yes | 1  2.115(1.760-2.542) | 0.000* |
| Urticaria  No  Yes | 1  1.321(1.017-1.717) | 0.037† |
| Rhinitis  No  Yes | 1  2.288(1.891-2.768) | 0.000* |
| Sleeping position |  |  |
| Supine | 1 | 0.019† |
| Lateral | 0.668(0.486-0.918) | 0.013† |
| Prone | 0.656(0.487-0.884) | 0.006* |
| Father`s sleep patterns |  |  |
| Very regular | 1 | 0.000* |
| Regular | 1.774(1.309-2.404) | 0.000* |
| Irregular | 3.014(2.170-4.187) | 0.000* |
| Mother`s sleep patterns |  |  |
| Very regular | 1 | 0.000* |
| Regular | 1.766(1.336-2.335) | 0.000* |
| Irregular | 3.467(2.445-4.917) | 0.000* |
| Father's bedtime |  |  |
| 21-22 | 1 | 0.000* |
| 22-24 | 1.629(1.307-2.031) | 0.000* |
| After 24 | 2.344(1.671-3.287) | 0.000* |
| Mother's bedtime |  |  |
| 21-22 | 1 | 0.000* |
| 22-24 | 1.401(1.162-1.691) | 0.000* |
| After 24 | 2.675(1.709-4.189) | 0.000* |
| Father's sleep duration |  |  |
| ＞8h | 1 | 0.001 |
| 6h-8h | 1.440(1.098-1.887) | 0.008 |
| ＜6h | 2.233(1.471-3.389) | 0.000 |
| Mother's sleep duration |  |  |
| ＞8h | 1 | 0.000 |
| 6h-8h | 1.406(1.126-1.756) | 0.003 |
| ＜6h | 3.156(2.003-4.975) | 0.000 |
| Familial sleep sickness | 2.630(2.164-3.198) | 0.000* |

P <0.05 was considered statistically significant difference. CI, confidence interval; OR, Odds ratios.
